# Cell wall structure and function in lactic acid bacteria

**DOI:** 10.1186/1475-2859-13-S1-S9

**Published:** 2014-08-29

**Authors:** Marie-Pierre Chapot-Chartier, Saulius Kulakauskas

**Affiliations:** 1 INRA, UMR1319 Micalis, F-78350 Jouy-en-Josas, France; 2 AgroParisTech, UMR Micalis, F-78350 Jouy-en-Josas, France

**Keywords:** Lactic acid bacteria, Cell wall, Peptidoglycan, Polysaccharide, Teichoic acid, Peptidoglycan hydrolase, Surface proteins, Autolysis, Bacteriophage, Probiotic, Bacteria-host cross-talk

## Abstract

The cell wall of Gram-positive bacteria is a complex assemblage of glycopolymers and proteins. It consists of a thick peptidoglycan sacculus that surrounds the cytoplasmic membrane and that is decorated with teichoic acids, polysaccharides, and proteins. It plays a major role in bacterial physiology since it maintains cell shape and integrity during growth and division; in addition, it acts as the interface between the bacterium and its environment. Lactic acid bacteria (LAB) are traditionally and widely used to ferment food, and they are also the subject of more and more research because of their potential health-related benefits. It is now recognized that understanding the composition, structure, and properties of LAB cell walls is a crucial part of developing technological and health applications using these bacteria. In this review, we examine the different components of the Gram-positive cell wall: peptidoglycan, teichoic acids, polysaccharides, and proteins. We present recent findings regarding the structure and function of these complex compounds, results that have emerged thanks to the tandem development of structural analysis and whole genome sequencing. Although general structures and biosynthesis pathways are conserved among Gram-positive bacteria, studies have revealed that LAB cell walls demonstrate unique properties; these studies have yielded some notable, fundamental, and novel findings. Given the potential of this research to contribute to future applied strategies, in our discussion of the role played by cell wall components in LAB physiology, we pay special attention to the mechanisms controlling bacterial autolysis, bacterial sensitivity to bacteriophages and the mechanisms underlying interactions between probiotic bacteria and their hosts.

## Introduction

The cell wall of Gram-positive bacteria is a complex arrangement of macromolecules. It consists of a peptidoglycan (PG) sacculus that surrounds the cytoplasmic membrane and that is decorated with other glycopolymers, such as teichoic acids (TAs) or polysaccharides (PSs), and proteins. The cell wall has multiple functions during bacterial growth, including maintaining bacterial cell integrity and shape as well as resisting internal turgor pressure. Furthermore, it must remain flexible to accommodate the remodeling that is required for cell division and growth. Since it serves as the interface between the bacterial cell and its environment, the cell wall also mediates bacterial interactions with abiotic surfaces, infecting bacteriophages, or eukaryotic host cells.

Lactic acid bacteria (LAB) are Gram-positive bacteria that belong to numerous genera, including *Lactococcus, Enterococcus, Oenococcus, Pediococcus, Streptococcus*, and *Lactobacillus *[1-3]. These bacteria metabolize sugars, mainly converting them to lactic acid, and are widely used as starters in the fermentation of food such as meat, vegetables, fruit, beverages, and milk. They play key roles in food preservation and contribute to the development of food texture and flavor [4,5]. Furthermore, LAB are present in the human gut microbiota. Certain natural LAB strains, lactobacilli strains in particular, are commercially sold as probiotics with health-promoting properties [6]. Finally, due to their GRAS (generally recognized as safe) status, LAB may be suitable vectors for the delivery of therapeutic proteins or antigens to mucosal surfaces [7,8].

When it comes to the technological and health applications of LAB, cell wall composition, structure, and component organization play major roles. The LAB cell wall has been the subject of research because it contains receptors for bacteriophages that threaten milk fermentation [9,10]. Research has also focused on the need to favor LAB cell wall disruption to provoke autolysis, so that, during cheese ripening, bacteria release their cytoplasmic content, which is rich in enzymes involved in the development of organoleptic properties [11]. It has also been suggested that increasing bacterial lysis by weakening the LAB cell wall can improve the efficiency of LAB as antigen-delivery vectors in immune system stimulation efforts [12]. More recently, it has been proposed that bacterial surface adhesins could favor the persistence of probiotic bacteria in the gastrointestinal tract [13]. Also, cell wall microbe-associated molecular patterns (MAMPs) identified in pathogens could play a role in the cross-talk that takes place between commensal or probiotic bacteria and their hosts [14,15]. As predicted by Delcour *et al*. [16], the availability of whole genome sequences has boosted research on LAB cell wall structure and function over the last fifteen years.

Here, we review the current state of knowledge on the structure and function of the cell wall components (PG, TAs, PSs, and proteins) of the most investigated LAB, including *Lactococcus lactis *and several lactobacilli, mainly *Lactobacillus plantarum, Lactobacillus casei*, and *Lactobacillus rhamnosus*.

## Peptidoglycan

### Chemical composition and structural analysis

**Figure 1:**
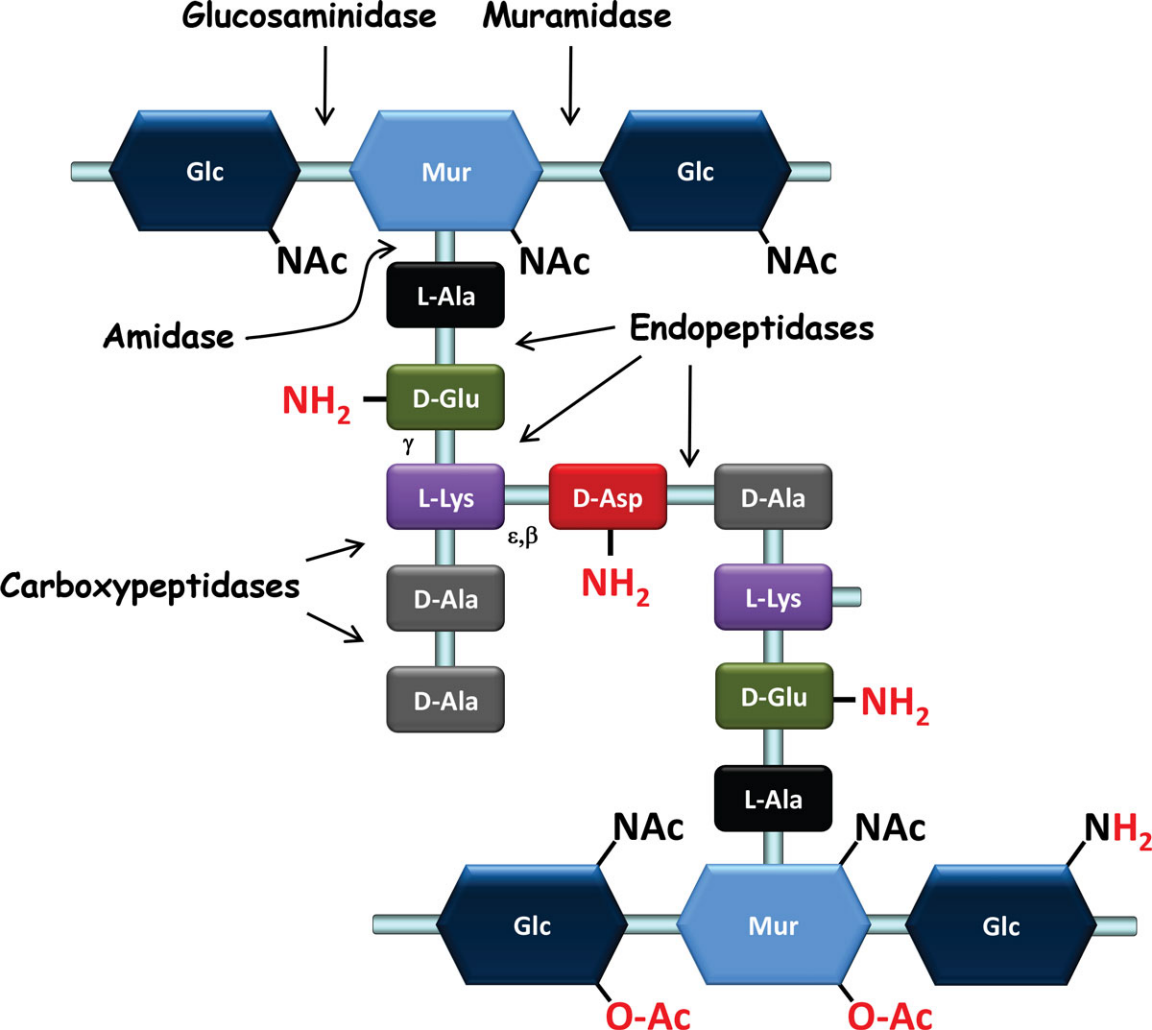
**Schematic representation of the structure of peptidoglycan**. This is the type of structure found in *L. lactis *and numerous lactobacilli. In other LAB species, the nature of the interpeptide cross-bridge (depicted as D-Asp/D-Asn in the figure) may vary, the third di-amino acid (L-Lys) may be replaced by mDAP or L-ornithine, and the D-Ala in position five of the stem peptide may be replaced by D-lactate. Possible modifications of the PG structure, such as *O*-acetylation (O-Ac), *N*-deacetylation (leading to GlcNH_2_), or amidation (NH_2_), are indicated in red. The cleavage sites of the different classes of PG hydrolases are indicated with arrows.

PG is the main component of the Gram-positive cell wall. It consists of glycan chains made of alternating *N*-acetylglucosamine (GlcNAc) and *N*-acetylmuramic acid (MurNAc) that are linked via β-1,4 bonds (Figure 1). Peptidic chains are linked covalently through their N-terminus to the lactyl group of MurNAc. These peptidic chains vary in composition across species and can be cross-linked directly or indirectly, through short chains of one or more amino acids that generate a three-dimensional structure around the cell, which ensures bacterial integrity. In LAB, the amino acid sequence of the stem peptide is L-Ala-γ-D-Glu-X-D-Ala, while the third amino acid (X) is a di-amino acid. It is most often L-Lys (e.g., in *L. lactis *and most lactobacilli) but can also be meso-diaminopimelic acid (mDAP) (e.g., in *L. plantarum*) or L-ornithine (e.g., in *L. fermentum*) [17]. Among LAB, D-Ala predominates at position five in newly synthesized PG; however, D-Lac residues are found in naturally vancomycin-resistant lactobacilli such as *L. casei *and *L. plantarum*. Cross-linking between neighboring stem peptides takes place between the D-Ala in position four of one peptide chain and the diamino acid in position three (4-3 cross-link) of another chain. A direct cross-connection is seen in mDAP-type PG, which is typically found in Gram-negative bacteria but which is also present in *L. plantarum*. In other LAB, the Lys-type PG is found and includes an interpeptide bridge made of one D-amino acid (e.g., D-Asp or D-Asn in *L. lactis, L. casei*, and most lactobacilli) (Figure 1) or several L-amino acids (e.g., L-Ala_2 _or L-Ala_3 _in *Streptococcus thermophilus*) [17]. PG peptide chains connected by 3-3 cross-links, which predominate in *Mycobacterium tuberculosis *[18] and in *Clostridium difficile *[19], have not been described in LAB to date.

Although a given bacterial species has a basic, characteristic PG structure, the PG layer remains in a dynamic state throughout a bacterium's life, and PG structure is the result of complex biosynthetic, maturation, and degradation reactions, which will be described below. Structural analysis of PG muropeptides using HPLC and mass spectrometry has allowed the identification of the nature of peptide cross-bridges, the degree of cross-linking, and the frequency of maturation and hydrolysis events. It has also revealed the existence of covalent PG modifications, such as *O*-acetylation, *N*-deacetylation, or amidation; these modifications may play essential roles in bacterial physiology. Detailed PG structure has been ascertained for several LAB, including *L. lactis *[20], *L. casei *[21], *L. rhamnosus *[22], and *L. plantarum *[23]. The first three species were found to have D-Ala^4^-D-Asp/Asn-L-Lys^3 ^cross-bridges, while the latter has a direct D-Ala^4^-mDAP^3 ^cross-bridge (Figure 1).

### Biosynthesis as a multi-step process

**Figure 2:**
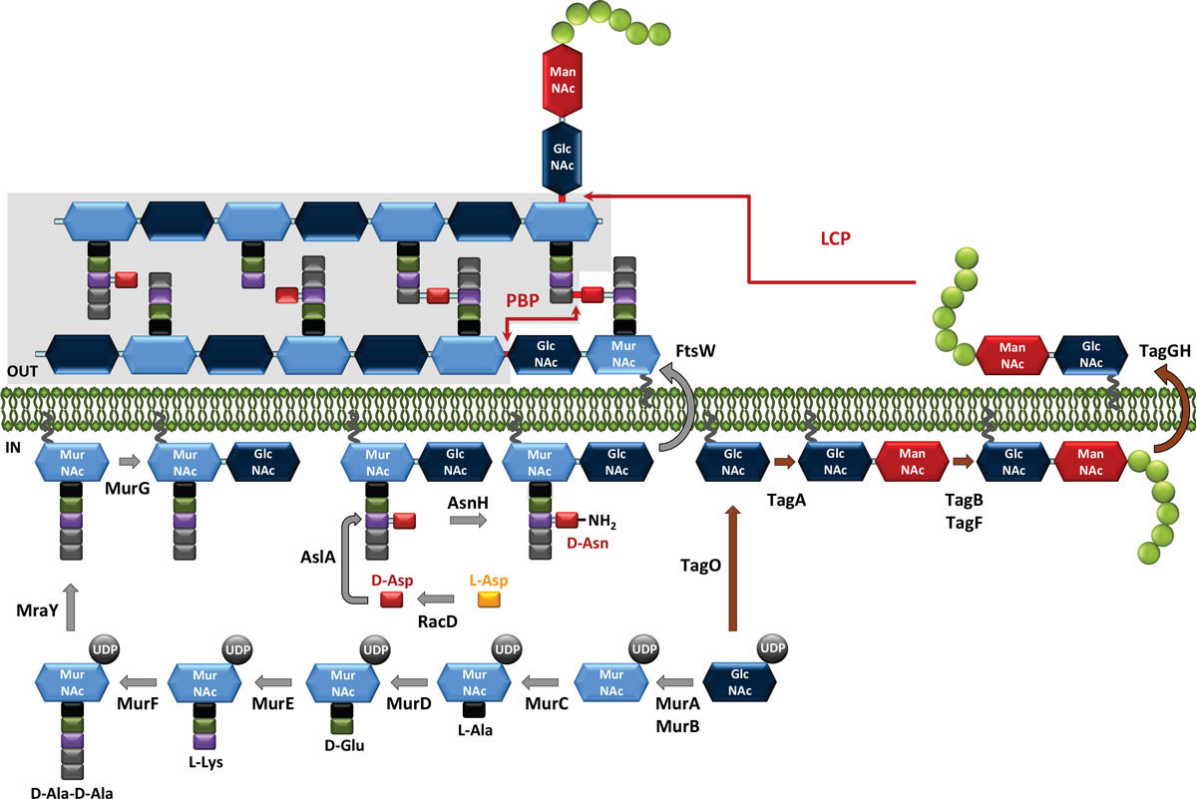
**Schematic representation of the main steps of peptidoglycan and wall teichoic acid biosynthesis**. Grey arrows denote the steps of PG biosynthesis, and brown arrows indicate the steps of WTA biosynthesis. The membrane-embedded undecaprenyl-phosphate carrier is represented by dark grey curved lines The glycerol-phosphate units are represented with green circles. The linkages formed by PBP and LCP are indicated with red arrows. The pre-existing PG is highlighted in gray. In the schematic, D-Asp is added to the lipid precursors; however, depending on the bacterial species, it may also be added to soluble precursors.

PG synthesis can be divided in three general steps: the first step takes place in the cytoplasm and leads to the synthesis of lipid II, the second step involves the transfer of lipid II to the extracellular side of the membrane, and the third step results in the polymerization of the synthesized subunits into a macromolecule [24] (Figure 2).

Assembly of lipid II starts with the synthesis of uridine diphosphate-*N*-acetyl glucosamine (UDP-GlcNAc) via the enzymatic conversion of glucosamine to energetically activated UDP-GlcNAc. UDP-MurNAc is then generated from UDP-GlcNAc, following two successive enzymatic reactions: the synthesis of enolpyruvate-UDP-GlcNAc and its subsequent reduction, which is catalyzed by MurA and MurB. Then, the UDP-MurNAc-pentapeptide precursor is assembled in a series of successive ATP-dependent enzymatic steps catalyzed by Mur ligases [25]. MurC and Mur D catalyze the addition of L-Ala and D-Glu, respectively, and MurE the one of L-Lys or mDAP. Finally, in a single step, MurF adds two residues in the form of a dipeptide (D-Ala-D-Ala) or a depsipeptide (D-Ala-D-Lac), whose synthesis requires D-D-ligases (Ddl). Specific racemases convert the naturally occurring L-stereoisomer of Ala and Glu to the D-forms found in PG [25]. In addition, in *L. plantarum*, which synthesizes precursors that terminate with D-Lac, D-Ala-D-Ala-dipeptidase (Aad) eliminates D-Ala-D-Ala dipeptides that are produced by the Ddl ligase, thereby preventing their incorporation into the precursors [26]. PG precursors terminating with D-Ala-D-Lac instead of with D-Ala-D-Ala were successfully produced in *L. lactis *when the *L. plantarum *Ddl ligase gene was heterologously expressed. Modification of the last residue of the stem peptides of PG precursors has been shown to result in significant changes to PG structure and cell morphology [27]. The UDP-MurNAc-pentapeptide is then attached with a pyrophosphate link to the lipid transporter, bactoprenol (undecaprenyl-phosphate), by the membrane translocase MraY, a process that yields undecaprenyl-pyrophosphoryl-MurNAc-pentapeptide, or lipid I (Figure 2). Finally, the glycosyl-transferase MurG adds GlcNAc to lipid I, forming undecaprenyl-pyrophosphoryl-disaccharide-pentapeptide, or lipid II, which is the basic subunit used in PG assembly [28].

Another important enzymatic step that takes place in the cytoplasm is the assembly of peptide side chains that are added either to the nucleotide MurNAc-pentapeptide or the lipid precursors, depending on the species [29]. D-Asp, the amino acid most commonly included in LAB side chains and that is found in *L. lactis *and in most lactobacilli, is added to the third amino acid (L-Lys) of the stem peptide by aspartate ligase (AslA) (Figure 2) [30]. This enzyme belongs to the ATP-Grasp family, which includes enzymes that catalyze ATP-dependent carboxylate-amine ligation reactions and that use activated D-Asp--in the form of β-aspartyl phosphate--as a substrate [31]. D-Asp is produced from L-Asp by the aspartate racemase encoded by *racD*, which is located in the same operon as the *aslA *gene in *L. lactis *[30,31]. The L-amino acids of the PG side chains are transferred from aminoacyl-tRNA by specific transferases, identified as BppA1 and BppA2 in *Enterococcus faecalis *[32], a species that has L-Ala-L-Ala cross-bridges like *S. thermophilus*.

Lipid II (with or without a side chain) is then translocated outside the cytoplasmic membrane by a flippase (Figure 2). The integral membrane protein FtsW has been shown to transport lipid-linked PG precursors across the membrane and is proposed to act at the septum level. The RodA homologous protein appears to be involved in lateral PG synthesis during cell elongation in ovococci and bacilli [33].

In the last step of PG synthesis, PG monomer units are polymerized via transpeptidation and transglycosylation reactions, which take place outside the cytoplasmic membrane (Figure 2). The major proteins involved in PG assembly are called penicillin-binding proteins (PBPs) because they are targets for penicillin and other beta-lactam antibiotics [34]. Class A PBPs contain both transglycosylation and transpeptidation domains located at the N- and C-terminals of the protein, respectively, whereas class B PBPs are exclusively involved in transpeptidation. During transglycosylation, lipid II's disaccharide is bound to the pre-existing PG chain; the bactoprenol loses one inorganic phosphate and is recycled to the inner side of the cytoplasmic membrane to initiate another round. To create a solid PG mesh around the bacterial cell, newly extended chains must be connected to neighboring chains by transpeptidation. A covalent bond is created between the carbonyl group of the D-Ala in position four of one pentapeptide chain (donor chain) and the free amine of either the diamino acid in position three of a second peptide chain or the attached side-chain amino acid (acceptor chain). This step leads to the release of the C-terminal D-Ala or D-Lac of the donor chain. Alternative 3-3 cross-links require L,D-transpeptidases, which are not PBPs [19,35].

Analysis of the genome of *L. lactis*, an ovococcus species, has revealed the presence of six PBPs: five high molecular weight (HMW) PBPs (PBP1a, PBP1b, PBP2a, PBP2b, and PBPx) and one low molecular weight (LMW) PBP (D-Ala-D-Ala-carboxypeptidase DacA) [36]. *L. lactis *also possesses an L,D-carboxypeptidase (DacB), which cleaves the L-Lys^3^-D-Ala^4 ^bonds of the stem peptides (Figure 1) [20]. Ovococci display both septal and peripheral growth, which results in the slight longitudinal expansion that generates their ovoid shape. It has been shown that lateral or septal growth is mediated by functionally different PG biosynthesis mechanisms, each under the control of a specific class B PBP: PBP2b and PBP2x, respectively. The other PBPs appear to have redundant functions, acting in both biosynthetic pathways [36]. Furthermore, alteration of PBP2x and PBP2b activity has been proposed to directly affect the coccus-to-rod transition and further filamentation observed in *L. lactis *during growth, both in planktonic conditions and biofilms [37].

Only part of the PG stem peptides are connected by transpeptidation, and the degree of cross-linking is a PG characteristic. During the exponential growth phase, the cross-linking index has been estimated to be 35.5% in *L. lactis*, 37.5% in *L. plantarum*, 34% in *L. casei*, and 36.5% in *L. rhamnosus *(Table 1). D,D-carboxypeptidase DacA and L,D-carboxypeptidase DacB participate in PG maturation and thus produce tetra-and and tripeptide chains in mature PG [20-23].

**Table 1:** Peptidoglycan structural variations in selected LAB and genes involved in PG synthesis or modification.

Peptidoglycan	*L. lactis* MG1363	*L. casei* BL23	*L. rhamnosus* GG	*L. plantarum *WCFS1
Cross-linking type	L-Lys-D-Asp	L-Lys-D-Asp	L-Lys-D-Asp	mDAP direct
Cross-linking index	35.5 %	34%	36.5 %	37.5%
*aslA gene*	**+**	**+**	**+**	**-**
*racD gene*	**+**	**+**	**+**	**+ **
Fifth residue stem peptide	D-Ala	D-Lac	D-Lac	D-Lac
Acetylation of MurNAc	3.2 %	30 %	37 %	39 %
*oatA *gene	**+**	**+**	**+**	**+**
Acetylation of GlcNAc	no	no	no	9 %
*oatB *gene	**-**	**-**	**-**	**+**
N-deacetylation of GlcNAc	9 %	no	no	no
*pgdA gene*	**+**	**+**	**+**	**-**
Amidation of D-Glu	100 %	100 %	100 %	100 %
*murT/gatD *genes	**+**	**+ **	**+ **	**+**
Amidation of D-Asp	75%	100 %	100 %	No D-Asp
*asnH *gene	**+**	**+**	**+**	**-**
Amidation of mDAP	No mDAP	No mDAP	No mDAP	100%
*asnB1 *gene	-	-	-	+

Another important feature of PG that likely influences PG architecture is glycan chain length. In *L. lactis*, long glycan chains (chains with more than 50 disaccharides, which represent 50% of all chains) were detected after an amidase treatment [38]. PG nanoscale architecture was examined using atomic force microscopy (AFM) in living *L. lactis *cells. When a mutant without PSs on its surface was imaged, using a tip functionalized with the PG-binding LysM domain, PG was found to be organized in the form of cables running parallel to the short axis of the cells [39].

### Peptidoglycan structural variations

In most bacterial species, PG basic structure is partially modified--either the glycan chains undergo *N-*deacetylation or *O*-acetylation or the free carboxyl groups of the amino acids in the peptide chains are amidated (Figure 1) (Table 1) [40]. These structural modifications usually have functional consequences (Table 2); for instance, they may modulate the activity of endogenous PG hydrolases (PGHs) as well as that of exogenous PGHs produced by eukaryotic organisms, such as lysozyme. PG modifications have been shown to allow pathogenic bacteria to escape from the host's innate immune system [41]. Below, we will review PG modifications by chemical groups, given that wall TA or PS polymers that covalently attach to PG may also be considered to be modifications; they can even be linked to the same sites on PG (see text below).

**Table 2:** Role of cell wall glycopolymers and their structural variations in LAB.

	Role	Organism*	**Ref**.
**PG variations**			

*O*-acetylation of MurNAc	Resistance to lysozyme Activation of autolysis by amidase LytH	*Ll, Lc, Lp* *Lp*	[23,45]
*O*-acetylation of GlcNAc	Inhibition of major autolysin Acm2 (glucosaminidase)	*Lp*	[23]
*N-*deacetylation of GlcNAc	Resistance to lysozyme Inhibition of autolysis Inhibition of AcmA major autolysin (glucosaminidase)	*Ll* *Ll* *Ll*	[54]
Amidation of D-Asp	Resistance to lysozyme Inhibition of autolysis Resistance to nisin.	*Ll* *Ll* *Ll*	[56]
Amidation of mDAP	Essential for growth Control of septation Increase DacB L,D-carboxypeptidase activity	*Lp* *Lp* *Lp*	[57]

**Teichoic acids**			

WTA	Bacterial morphogenesis and division	*Lp*	[120]
LTA	Immunomodulatory properties	*La*	[127]
TA alanylation	Cell morphology UV stress response Protein secretion Resistance to nisin Bacteriophage receptor Adhesion to epithelial cells Decrease of anti-inflammatory properties Colonization of mouse gastrointestinal tract	*Lp, Lrh* *Ll* *Ll* *Ll, Lp* *Ld* *Lj* *Lp, Lrh* Lr	[85,112] [114] [113] [52,85,111] [122] [123] [100,126] [124]

**Polysaccharides**			

	Cell division and morphology Bacteriophage receptor Protection against phagocytosis	*Ll* *Ll* *Ll*	[131] [131,133,144] [131]
	Immunosuppressive function	*Lc, Lp*	[136,142]
	Decrease adhesion and biofilm formation Protection against antimicrobial peptides (LL-37)	*Lrh* *Lrh*	[137] [147]

* *Ll, Lactococcus lactis; Lc, Lactobacillus casei; Lp, Lactobacillus plantarum; Lrh, Lactobacillus rhamnosus; La, Lactobacillus acidophilus; Ld, Lactobacillus delbruekii subsp. lactis; Lj, Lactobacillus johnsonii; Lr, Lactobacillus reuteri*.

#### O-Acetylation of glycan chains

In many Gram-positive pathogens, *O*-acetylation of MurNAc is associated with resistance against lysozyme [42]. A certain proportion of MurNAc residues have an extra acetyl group linked to their C6-OH that can be used to form a 2,6-N,O-diacetyl muramic acid (Figure 1). The first MurNAc *O*-acetyltransferase, named OatA, was identified in *Staphylococcus aureus *[43]. OatA is conserved among a large number of Gram-positive species, including LAB [23,44,45]. The enzyme is composed of two domains: the N-terminal domain contains 11 predicted transmembrane helices, whereas the C-terminal domain appears to contain a catalytic acetyltransferase domain. The donor of the acetyl group is probably acetyl-CoA [46]. The acetyl group is likely added to the newly polymerized PG outside the cytoplasmic membrane since *O*-acetylation of lipid precursors has not been observed [43] and the OatA acetyltransferase domain is predicted to be located outside the membrane. *O*-acetylation of MurNAc residues has been detected in the different LAB species for which structural analysis of PG has been performed; estimated levels of *O*-acetylation vary, from rather low in *L. lactis *MG1363 (3.2%) [20] to intermediate in lactobacilli: *L. casei *BL23 (30%) [21], *L. plantarum *NZ7100 (39%) [23], and *L. rhamnosus *GG (37%) (Table 1) [22].

In *L. lactis*, the *oatA *gene has been shown to be regulated at the transcriptional level in response to cell envelope stress, which may be provoked by lysozyme or other cell wall-targeting antimicrobials such as bacitracin, vancomycin, and plantaricin [45,47]. It has been proposed that the first lactococcal response to treatment with lysozyme is the activation of the two-component system (TCS) CesSR, which then activates the transcription of several genes belonging to the *cesSR *regulon, among which is *spxB *[47], which belongs to the family of global transcriptional factors found in Gram-positive bacteria [48]. SpxB activates *oatA *expression; OatA activity increases PG resistance to lysozyme and thus counteracts cell wall stress [45]. Interestingly, while increased PG *O*-acetylation makes *L. lactis *more resistant to the PGH activity of lysozyme, it has been shown that the *cesSR *regulon is induced by overexpression of membrane-anchored proteins [49,50] or by bacteriophage infection [51]. The regulon has also been shown to be induced in an *L. lactis *mutant resistant to the bacteriocin nisin [52].

The *O*-acetylation of GlcNAc, never before described in bacteria, was discovered in *L. plantarum*; both GlcNAc and MurNAc are acetylated in this species [23]. In this bacterium, around 9% of GlcNAc residues are *O*-acetylated. The addition of the acetyl group to GlcNAc is performed by a second, specific *O*-acetyltransferase--OatB--that shares a similar two-domain structure with *L. plantarum *OatA but has a rather low amino acid sequence identity (21%). It is noteworthy that, until now, the presence of two Oat proteins has only been found in a very limited number of bacterial species, including two other LAB species, *Lactobacillus sakei *and *Weissella paramesenteroides *[23].

PG *O*-acetylation has an impact on *L. plantarum *autolysis. The *O*-acetylation of GlcNAc inhibits the *N*-acetylglucosaminidase Acm2, the major autolysin of *L. plantarum*. In contrast, in this species, *O*-acetylation of MurNAc has been shown to activate autolysis through the activity of the putative *N*-acetylmuramoyl-L-alanine amidase LytH [23]. Thus, both *O*-acetyltransferases, OatA and OatB, which co-occur in *L. plantarum*, play antagonistic roles when modulating the activity of endogenous autolysins. In contrast to the *O*-acetylation of MurNAc, the *O*-acetylation of GlcNAc does not inhibit lysozyme activity [23].

#### N-Deacetylation of glycan chains

The *N*-deacetylation of GlcNAc, which leads to the presence of glucosamine (GlcNH_2 _on Figure 1) in the PG backbone, is performed by PG-deacetylase PgdA, which was first identified in *Streptococcus pneumoniae *thanks to its sequence homology with chitin deacetylases [53]. GlcNAc deacetylation has been found to occur at a level of around 9% in *L. lactis*; in this species, it protects PG from hydrolysis by AcmA autolysin [54] and increase resistance to lysozyme [45]. In contrast, the *N*-deacetylation of GlcNAc has not been observed in *L. casei, L. rhamnosus*, or *L. plantarum *under laboratory growth conditions. A *pgdA *homolog is present in the *L. casei *BL23 genome, while no homolog exists in the *L. plantarum *genome (Table 1). Deacetylated MurNAc residues were found in *Bacillus anthracis *PG [40] and, recently, a MurNAc-deacetylase was discovered in *Bacillus subtilis *[55]; neither have been found in LAB to date.

#### Amidation of amino acids

The free carboxyl groups of PG-forming amino acids can be amidated; these amino acids include D-Glu and mDAP found on stem peptides and D-Asp on side chains or cross-bridges (Figure 1). These modifications are catalyzed by specific enzymes and take place intracellularly; PG precursors, either UDP-MurNAc-pentapeptide or lipid intermediates, are amidated before the molecules are translocated through the cytoplasmic membrane [29]. Amidation of D-Asp cross-bridges has been observed in *L. lactis *[20]. D-Asn and D-iso-Asn are not substrates for aspartate ligase, as has been shown in *L. lactis *and *Enterococcus faecium*, a species that also has D-Asp cross-bridges [30,31]. As a result, amidation of the alpha-carboxyl group of D-Asp takes place after D-Asp has been added to the PG precursor and is performed by an asparagine synthase (AsnH), which was identified in *L. lactis *[56] (Figure 2). In *L. lactis*, amidation of the D-Asp cross-bridge during the exponential phase is partial (75%); in contrast, in *L. casei*, it is almost complete (near 100%) during all growth phases. PGH activity is affected by D-Asp amidation. Indeed, an *L. lactis asnH *mutant with PG that contained exclusively D-Asp bridges exhibited a higher autolysis rate than the wild-type strain, as well as increased sensitivity to lysozyme. D-Asp amidation also decreases *L. lactis *sensitivity to cationic antimicrobials such as nisin, which may be explained by a decrease in the negative charge inside the cell wall [56].

In *L. plantarum*, almost all the mDAP side chains are amidated. In this bacterium, amidation has also been shown to be mediated in the cytoplasm by an amidotransferase named AsnB1, the first enzyme to be associated with such activity [57]. Interestingly, the *asnB1 *gene co-localizes with *murE*, which encodes the ligase catalyzing the addition of mDAP to the PG precursor UDP-N-muramoyl-L-Ala-D-Glu. The *asnB1 *gene has been found to play an essential role in *L. plantarum*. In a mutant strain with a mDAP amidation defect, growth and cell morphology were strongly affected; filamentation and long-chain formation were observed, suggesting that mDAP amidation may play a critical role in controlling the septation process. In addition, L-D-carboxypeptidase DacB activity requires mDAP amidation to reach optimal levels [57].

The D-Glu on the PG stem peptide has an amidated α-carbonyl group (which transforms it into an iso-Gln) in several bacterial species, including LAB. The level of amidation is close to 100% in all four of the LAB species studied [20-23]. The genes responsible for D-Glu amidation have been identified in *S. aureus *[58,59]. The conversion of iso-Glu to iso-Gln is catalyzed by the glutamine amidotransferase GatD and the Mur ligase homolog MurT. Lipid precursors, but not soluble UDP-MurNAc-pentapeptide precursors, are substrates for this enzymatic complex [59]. The *murT *and *gatD *genes are grouped in an operon and play an essential role in *S. aureus*. Past research has found that inhibition of amidation results in a markedly reduced bacterial growth rate, which suggests that amidated PG may serve as a better substrate for proteins that catalyze PG biosynthesis and cell division; furthermore, resistance to beta-lactam antibiotics and increased sensitivity to lysozyme have been observed [58]. Homologs of co-localized *gatD *and *murT *genes are present in LAB with amidated D-Glu PG including *L. lactis and L. plantarum; *in *L. casei *and *L. rhamnosus*, both genes can be found but at different places in the genome sequences (Table 1).

### Degradation by PGHs

PGHs are enzymes that can hydrolyze specific bonds in bacterial cell wall PG. Among them are bacterial autolysins and phage endolysins. Autolysins are endogenous bacterial PGHs whose activity may lead to autolysis, in particular when cells experience stressful conditions. Moreover, the cleavage of PG strands is required to insert newly synthesized PG subunits during bacterial cell growth and to separate daughter cells following cell division [60,61]. Bacteriophage genomes encode PGHs called endolysins that, in association with holins, are responsible for host cell destruction after the viral particles have multiplied during phage dissemination [62]. They may also encode PGHs that are tail-associated lysins involved in phage entry into the host bacteria [63]. From a technological cheese making point of view, highly focused, applied studies have sought to understand and control LAB lysis, with the aim of being able to release the intracellular pool of enzymes of starter bacteria to improve cheese flavor development [11,64].

Different classes of PGHs can be defined on the basis of their hydrolytic specificity for different bonds (Figure 1): (i) *N*-acetylmuramidases (muramidases) hydrolyze the β1-4 bond between MurNAc and GlcNAc--among the known muramidases are lysozymes that result in a product with a terminal-reducing MurNAc residue and lytic transglycosylases that yield anhydromuropeptides as a result of the formation of a 1,6-anhydro ring inside MurNAc; (ii) *N*-acetylglucosaminidases (glucosaminidases) hydrolyze the β1-4 bond between GlcNAc and MurNAc; (iii) *N*-acetylmuramyl-L-Ala amidases (amidases) hydrolyze the bond between the lactyl group of MurNAc and the α-amino group of L-Ala, which is the first amino acid of the lateral peptidic chain; and (iv) peptidases, including endopeptidases and carboxypeptidases, hydrolyze a variety of PG bonds.

Bacterial PGHs as well as phage endolysins usually exhibit modular organization and have a catalytic domain associated with a cell wall binding domain (CWBD). The catalytic domain determines hydrolytic specificity for the PG molecule, whereas the CWBD, because it specifically recognizes a cell wall component, influences localization, target bacteria specificity, and/or PGH catalytic efficiency. CWBDs, such as the LysM domain, the SH3 domain, or the Lc-LysBD domain, that are found in PGHs but also possibly in other cell wall proteins are described in more detail in the text below.

The catalytic domains present in LAB PGHs are characteristic of the following enzyme families: the glucosaminidases (glycoside hydrolase family 73, PF01832), the muramidases (glyco_hydro_25, PF01183), the lytic transglycosylases (transglycosylase-like, PF06737), the amidases (two domains: Amidase_2, PF01510 and Amidase_3, PF01520), the CHAP-domain enzymes with amidase or endopeptidase specificity (the cysteine, histidine-dependent amidohydrolase/peptidase domain) (PF05257), and the γ-glutamyl-diamino-acid endopeptidases (NlpC_P60, PF00877). Furthermore, the Peptidase_S11 domain (PF00768) is present in D,D-carboxypeptidases (DacA) and the VanY domain (PF02557) is found in L,D-carboxypeptidase (DacB).

The availability of complete genome sequences allows the full PGH complement of a given bacterial species to be analyzed and identified using amino acid sequence similarity searches that employ representative sequences of all known classes of PGHs. Most Gram-positive bacteria possess a complex PGH complement that includes a variable number of PGHs. Generally, a given bacterial species produces several PGHs that have various hydrolytic specificities, although not necessarily all the specificities listed above. In LAB, sequence analyses have revealed that rather complex PGH systems exist; 12 PGHs were identified in *L. casei *[21], 9 in *L. helveticus *[65], and 12 in *L. plantarum *[66]. Five PGHs were initially identified in *L. lactis*, before the description of the CHAP domain [67]; re-examination of the genome sequence of *L. lactis *MG1363 has allowed us to identify 4 additional putative PGHs that contain a CHAP domain (unpublished results).

Before whole genome sequencing, the first LAB PGH characterized at the molecular level was the major autolysin AcmA in *L. lactis *[68]. AcmA has a modular structure; its N-terminal catalytic domain demonstrates *N*-acetylglucosaminidase specificity [69], and its C-terminal domain is made up of three LysM sequences. The LysM repeats have been shown to bind to PG, and binding appears to be hindered by other cell wall constituents, which results in localized binding of AcmA to the cellular septum [70]. In *L. plantarum*, the major autolysin Acm2 is also an *N*-acetylglucosaminidase, but its modular structure differs from that of AcmA. In addition to its catalytic domain, it has three SH3 domains and an *N*-terminal Ala/Ser/Thr (AST)-rich domain [66,71]. Two other major PGHs with γ-D-Glu-L-Lys-endopeptidase activity, Msp1 (p75) and Lc-p75, have been characterized in *L. rhamnosus *[22] and *L. casei *[21], respectively. In contrast, Cse in *S. thermophilus*, which has a CHAP domain, has demonstrated D,L-endopeptidase activity and mediates cleavage of the PG cross-bridge [72]. It is worth noting that these enzymes are, respectively, the major autolysins of the aforementioned bacterial species and they are involved in daughter cell separation. They illustrate the diversity that exists among bacterial species in cell-separating enzymes, a point highlighted in past research [73]. In each species, inactivation of the corresponding genes led to defects in daughter cell separation and long-chain formation. In agreement with their role, all these PGHs were located at the cell septum. Also, AcmA and Acm2 are the major autolysins involved in the bacterial cell autolysis that is observed during the stationary phase or after bacteria are transferred to buffer solution. Other PGHs have been characterized in *L. lactis*: three other glucosaminidases, one with a LysM domain (AcmD) and two without LysM domains (AcmB and AcmC) as well as one γ-D-Glu-L-Lys-endopeptidase, YjgB [74]. AcmB and AcmD contribute to autolysis, in tandem with AcmA [75,76] and AcmD but not AcmB, is involved in cell separation [76]. In *L. plantarum*, LytA with putative γ-D-Glu-L-Lys-endopeptidase activity, appears to be required for cell shape maintenance and cell wall integrity [66].

Interestingly, several LAB PGHs have recently been shown to be *O*-glycosylated [71,77,78]. Their sugar residues are covalently linked to low complexity domains rich in Ala, Ser, and Thr (AST domains). *L. rhamnosus *p75 (Msp1) has been found to be glycosylated with hexoses, and probably mannose, given that they are recognized by the lectin concanavalin A. *O-*glycosylation of p75 (Msp1) appears to confer protection against proteolytic degradation [77]. Furthermore, *L. plantarum *Acm2 contains more than 20 bound N-acetyl-hexosamines: most are probably GlcNAc residues given that they are recognized by wheat germ agglutinin [78]. In this species, *O*-glycosylation has been shown to modulate Acm2 PG-degradation activity (see section 1.5) [71]. Very recently, the glycosyltransferases involved in Acm2 *O*-glycosylation were identified [79].

### Factors controlling PG hydrolysis

A strong PG mesh is needed to maintain cell shape and to counteract both high turgor pressure and cell wall stress related to environmental factors. At the same time, the growth and separation of bacterial cells also require a high degree of PG elasticity. These two opposing demands require the coordinated and balanced action of PG synthetic and degradation enzymes. The loss of this equilibrium may cause growth arrest and cell lysis. In bacteria, such equilibrium is achieved mostly by regulating activities of potentially lethal autolytic enzymes that are PGHs. PGH regulation can take place at the transcriptional level but may also be mediated by mechanisms involving post-transcriptional modifications of PGHs or modification of their substrate, PG [60,61,80].

#### Control of PGH activity

One of the factors that affects autolysin activity is proteolytic degradation. It has been shown that the main lactococcal autolysin, AcmA, is degraded by extracellular proteinase PrtP and that the autolysis of *L. lactis *MG1363 depends on the expression of the plasmid encoded cell wall-anchored proteinases PrtPI and PrtPIII [81]. Also, the cell wall-housekeeping protease HtrA has been shown to process lactococcal autolysin AcmA [82].

The activity of a given PGH can also be affected by its specific location in the bacterial cell. Depending on their role in bacterial physiology, PGHs may be distributed all along the cell periphery or located at the septum, as has been observed for PGHs involved in daughter cell separation. By immunofluorescent labeling, the major LAB autolysins (*L. lactis *AcmA, *L. casei *Lc-p75, *L. rhamnosus *p75*, S. thermophilus *Cse, and *L. plantarum *Acm2) were found to be localized in the septal zone of dividing cells [21,22,70-72].

Secondary cell wall polymers (TAs or PSs) can modulate autolytic activity by shielding PG [83]. In *L. lactis*, secondary cell wall polymers can hinder the binding of AcmA LysM domain to PG, which results in the localized binding of AcmA [70]. Furthermore, the autolysis of LAB strains can be influenced by the level of D-alanylation of TAs. An *L. lactis dltD *mutant, deficient in LTA alanylation, exhibited increased autolysis, which was tied to the decreased degradation of AcmA by HtrA protease [84]. The absence of D-Ala on LTAs in *L. plantarum *increases autolysis, caused, at least partially, by the autolysin Acm2 [85].

Also, as described above in the text above, structural variations in the PG substrate, such as the *O*-acetylation of glycan chains and the amidation of peptide chains, can contribute to the modulation of PGH activity.

Finally, glycosylation of the autolysin Acm2 has recently been shown to control the enzyme's activity [71]. The N-terminal AST-rich domain of Acm2 is glycosylated; this domain bears 21 mono-GlcNAc that are linked to Ser or Thr residues. When the AST domain is not *O*-glycosylated, Acm2 enzymatic activity significantly increases. In the model that has been proposed, the access of the Acm2 catalytic domain to its substrate may be hindered by the AST domain; *O*-glycosylation could change the domain conformation and/or mediate interdomain interactions [71].

#### Regulation at the transcriptional level

Most studies have focused on the transcriptional regulation of the genes encoding endogenous PGHs in the context of the cell envelope stress response [86]. It has been shown that the expression of PGH genes is positively regulated by WalRK TCS in *S. aureus *[87] and by the alternative sigma factor D in *B. subtilis *[88]. Interestingly, instead of affecting the transcription of genes that encode endogenous PGHs, the lactococcal TCS that responds to cell envelope stress, CesSR, increases expression of the *oatA *gene, and this gene encodes PG *O*-acetyltransferase, whose activity increases PG resistance to the PGH lysozyme [45] (see text above).

### PG and PGH as mediators of bacteria-host interactions

PG and certain of its fragments are known MAMPs that are recognized by host pattern-recognition receptors (PRRs), such as Nod receptors or Toll-like receptors (TLR) [89]. Nod receptors are intracellular receptors expressed by both epithelial cells and immune cells, such as dendritic cells. The minimum ligand recognized by Nod1 is the dipeptide D-Glu-mDAP, which is present in most Gram-negative bacteria, and the minimum ligand recognized by Nod2 is MurNAc-L-Ala-D-Glu, which is present in most bacteria [90]. In addition to the activity of host PGHs such as lysozyme and certain PG-recognition proteins (PGRP), endogenous bacterial PGHs may contribute to the release of PG fragments that can modulate host response [80]. For example, the PG of an *L. casei *mutant contained less disaccharide-dipeptide (GlcNAc-MurNAc-L-Ala-D-Gln), a known Nod2 agonist; this mutant lacked the major PGH Lc-p75, which demonstrates γ-D-Glu-L-Lys-endopeptidase activity [21], a fact that possibly affected Nod2 signaling. When the PG structures of two *Lactobacillus *strains with different inflammation profiles were compared, the presence of the muropeptide MurNAc-L-Ala-D-Glu-L-Lys (M-tri-Lys) in the PG structure and the anti-inflammatory properties of *Lactobacillus salivarius *Ls33 were found to be correlated [91]. The corresponding synthetic muropeptide has been shown to have a protective effect in a mouse model of intestinal inflammation (Nod2-dependent). These results show that PG originating from probiotic or commensal LAB may play an active role in the gut's immune balance.

Furthermore, in the well-documented probiotic *L. rhamnosus *GG, two secreted PGHs, Msp1 (p75) and Msp2 (p40), were found to promote the survival and growth of epithelial cells under pro-inflammatory conditions [92]. Furthermore, p40 has been shown to prevent and treat colonic epithelial cell injury and inflammation in mouse models of colitis through a mechanism that is dependent on the epidermal growth factor (EGF) receptor [93]. The non-catalytic N-terminal domain, which does not contain any characterized functional domains, appears to be responsible for the beneficial effects [94].

## Teichoic acids

### Structures of teichoic acids

The cell wall of most Gram-positive bacteria contains TAs, which are anionic polymers made of alditol-phosphate repeating units [95]. They are classified into two groups: wall teichoic acids (WTAs), which are covalently linked to the PG molecule, and lipoteichoic acids (LTAs), which are anchored in the cytoplasmic membrane with a glycolipid moiety. WTAs may constitute up to half of cell wall total dry weight in certain bacterial species [96].

**Figure 3:**
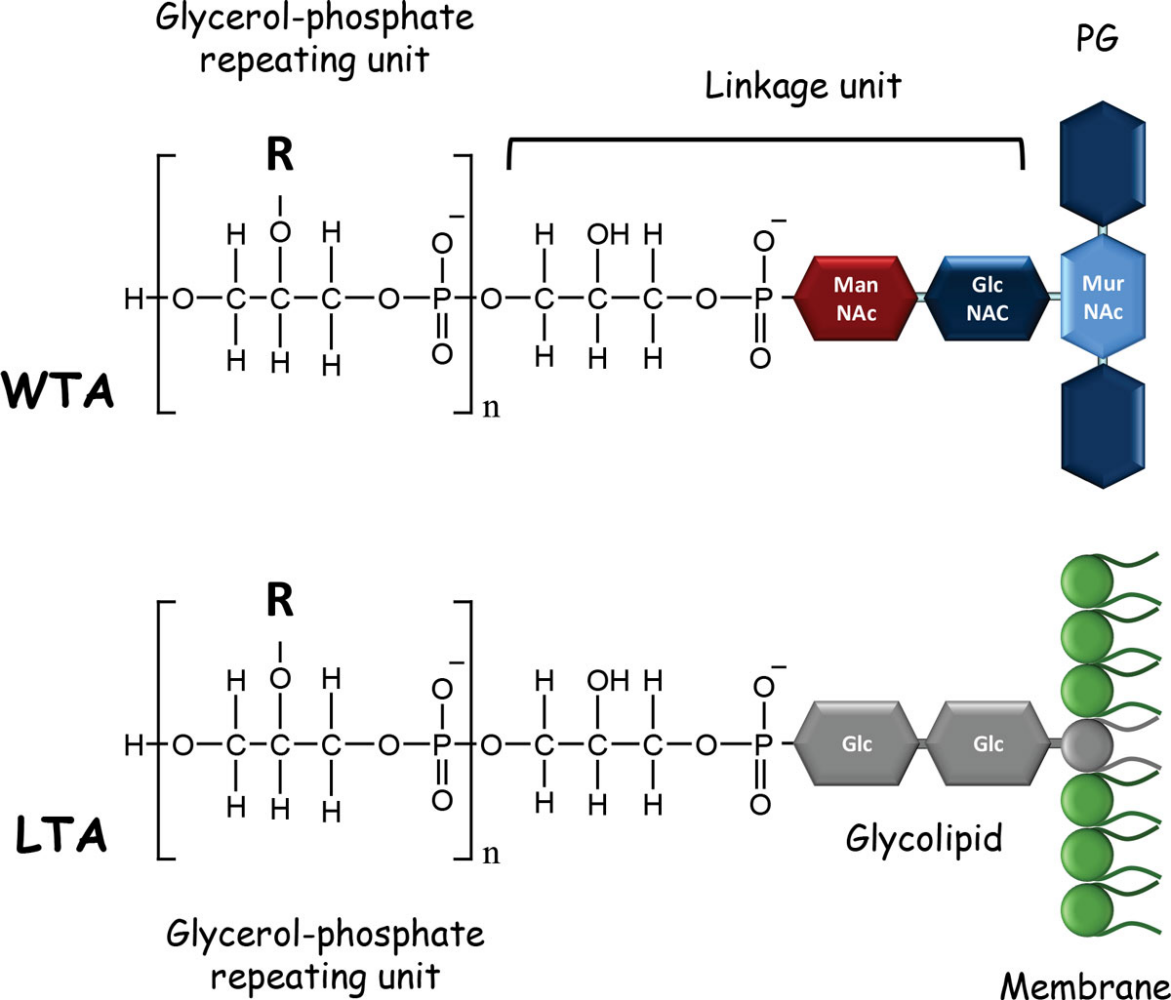
**Schematic representation of wall teichoic acid (WTA) and lipoteichoic acid (LTA) structure**. WTAs and LTAs with poly(glycerolphosphate) chain structure are represented. WTAs are covalently bound to PG by a phosphodiester linkage to the C6-hydroxyl of MurNAc via a linkage unit that usually consists of a disaccharide and a glycerol-phosphate unit. LTAs are anchored to the cytoplasmic membrane by a glycolipid (indicated in gray), which is a diglucosyldiacylglycerol. The R- indicates the substituents (e.g., D-Ala, Glc, Gal, or GlcNAc) found on the glycerol-phosphate chains.

The most common WTA structures are poly-glycerophosphate [poly(Gro-P)] or poly-ribitolphosphate [poly(Rbo-P)] chains. They are covalently attached to PG by a phosphodiester linkage to the C6-hydroxyl of MurNAc, via a linkage unit usually consisting of a disaccharide, N-acetylmannosaminyl-β(1-4)-N-acetylglucosamine, and a Gro-P unit (Figure 3). The typical LTA structure consists of a poly(Gro-P) chain linked to a glycolipid anchor (Figure 3). The free hydroxyl groups on the Gro- or Rbo-alditol units are partly decorated with D-Ala or monosaccharides, such as Glc, Gal or GlcNAc. The length of the poly(Gro-P) or poly(Rbo-P) chains varies between species and strains, as does the substitution level. In most Gram-positive bacteria, LTAs and WTAs coexist, but certain bacterial species, including *L. casei *and *L. rhamnosus*, appear to contain only LTAs. Remarkably, in *L. plantarum *(depending on the strain), the two types of WTAs--with either poly(Gro-P) or poly(Rbo-P) chains--have been found [97]; in addition, certain strains contain the genes needed to synthesize the two types of WTAs [98]. In an *L. plantarum *mutant in which the Gro-P type WTA synthesis was abolished, an alternative ribitol-type WTA was synthesized instead of the wild-type Gro-P type WTA [99]. LTA purified from *L. rhamnosus *and *L. plantarum *were analyzed by NMR; they were found to have poly(Gro-P) backbones containing an average of 50 and 22 Gro-P repeating units (for each species, respectively), with D-Ala being the only detectable substituent (74% and 42% D-Ala/GroP, respectively) [100,101]. *L. lactis *was found to have poly(Gro-P) LTAs with D-Ala and Gal substituents [52].

### Biosynthesis of WTAs and attachment to PG

Depending on the nature of the alditol constituent, biosynthetic enzymes are named Tag (for Gro-containing WTAs) or Tar (for Rbo-containing WTAs) [102,103]. We present here the synthesis of Gro-containing WTAs, which has mainly been described for *B. subtilis*; however, a similar biosynthesis scheme was found for Rbo-containing WTAs present in *S. aureus*. The first step is initiated on the cytoplasmic face of the membrane by TagO, an enzyme that transfers GlcNAc-1-P from UDP-GlcNAc to undecaprenol phosphate, the lipid carrier also involved in PG synthesis (Figure 2). Then, acetylmannosamine (ManNAc) is transferred from UDP-ManNAc by TagA. UDP-ManNAc is synthesized by the epimerization of UDP-GlcNAc to UDP-ManNAc by MnaA. The synthesis of the linkage unit is finished when TagB primase sequentially attaches one Gro-P unit taken from CDP-glycerol. CDP-glycerol is formed from CTP and glycerol by TagD. WTA polymerization is catalyzed by TagF, which adds Gro-P taken from CDP-glycerol to the nascent WTA chain. Up to 60 alditol-phosphate groups may be successively added. After the intracellular steps are completed, the WTA chain is translocated to the extracellular side of the membrane by the ABC transporter TagGH; subsequently, the chain is covalently linked to PG--on the C6-OH of MurNAc. Three transferases (TagTUV) belonging to the LytR-CpsA-Psr (LCP) family involved in the transfer of the bactoprenol-linked neo-synthesized WTAs to PG have been identified in *B. subtilis *[104]. Homologous enzymes are also found in LAB such as *L. lactis*, but their role has yet to be investigated.

### Biosynthesis of LTAs and their anchoring in the cytoplasmic membrane

LTAs are linked to the bacterial cell by a glycolipid inserted in the outer layer of the membrane (Figure 3). This glycolipid is a diglucosyldiacylglycerol synthesized from diacylglycerol by the successive addition of two Glc from UDP-Glc by YpfP [105]. LtaA then transfers the diglucosyldiacylglycerol from the inner to the outer side of the membrane. Once outside the membrane, the diglucosyl portion of the lipid anchor is elongated by LtaS via a polymerization process that adds, in most species, Gro-1-P units; the resulting LTA chain can contain up to fifty such units. In certain species, the first unit is added by a specific LTA-primase (LtaP), the role of which is to initiate elongation. The donor of Gro-P is a phosphatidyldiacylglycerol molecule. The diacylglycerol that is released can be recycled to synthesize another LTA molecule.

### Modifications of teichoic acids

As mentioned above, the free hydroxyl groups of the alditol-phosphate chains of both the WTAs and the LTAs may be replaced by different sugars (e.g., Glc, Gal, or GlcNAc) or by D-Ala. In *B. subtilis*, a glycosyltransferase named TagE adds Glc to WTAs [106]. The D-alanylation process is the best characterized and involves the *dltABCD *operon [107]. The first step is the activation of D-Ala, which consumes ATP, and the alanylation of the DltC carrier by DltA. Two models have been proposed for the next steps involving DltB and DltD [108,109]. In the first model [110], DltB transfers D-Ala from DltC to undecaprenol-phosphate lipid (C55-P) and is then flipped outside the membrane. DltD is then involved in transfer of D-Ala to LTA. In the second model [107], DltD would rather act in the cytoplasm by promoting transfer of D-Ala to DltC. The DltC-Ala is then transported through the membrane by the protein DltB. DltC would be the only protein required for the transfer of D-Ala to LTA. Recent data rather substantiates the first model although a lipid-linked intermediate has not been detected until now [109]. It seems that D-Ala residues are then free to move along a single alditol-phosphate chain or between different chains, which allows the D-alanylation of WTAs [107]. D-alanyl substituents can modulate the net negative charge of teichoic acids by providing protonated amino groups that serve as counterions to the negatively charged phosphate groups. These modifications widely contribute to TA functionality (see text below).

A *dlt *operon has been identified in the different LAB that have been studied. In *L. plantarum*, the *dlt *operon contains two supplementary genes, *pbpX2 *and *dltX*, that encode, respectively, a protein whose sequence is similar to that of a low molecular weight PBP endowed with D,D-carboxypeptidase activity and a small protein of 49 amino acids in length whose function is unknown [85]. When the *dlt *operon is inactivated, D-Ala substituents on teichoic acids are completely absent or strongly reduced in number, a result that has been observed in *L. lactis, L. rhamnosus*, and *L. plantarum *[84,85,111,112]. Unexpectedly, D-Ala-depleted LTAs in the *L. plantarum *NCIMB8826 *dltB *mutant contained high levels of Glc subtituents that were absent from wild-type LTAs and that were threefold longer than wild-type LTAs [100]. Also, in *L. rhamnosus *GG, LTA extracted from a *dltD *mutant revealed longer fatty acid chains of the glycolipid anchor, and shorter chain of Gro-P compared to the wild-type LTA [112].

### Functions of teichoic acids

WTAs and LTAs contribute significantly to cell wall functionality (Table 2). The various roles attributed to TAs are, at least in part, related to their anionic character or their distribution within the bacterial cell wall. The level of D-Ala substituents, which modify the global and local charge of TAs, also has a major impact on their functionality [107].

In general, TAs provide a reservoir of ions close to the cell wall that may be required for enzymes to function properly. Due to their anionic character, they can bind both cations, such as Mg^2+^, and protons, thus creating a pH gradient across the cell wall [107]. They play other roles: they control autolysins, maintain bacterial cell morphology, recognize bacteriophages, interact with the host immune system, and are involved in host colonization. These roles are detailed below. In *L. lactis*, LTA D-alanylation also has an impact on the efficiency of protein secretion [113], UV stress resistance [114], and resistance to the cationic antimicrobial peptide nisin [52].

#### TA and autolysis control

TAs and their substituents have long been considered to play a role in the control of bacterial autolysis in certain Gram-positive bacterial species; they are thought to act using several proposed mechanisms. LTAs were initially considered to be autolysin inhibitors. By determining the number of binding sites for cationic autolysins, their degree of D-alanylation has been also proposed to be a means of regulating autolysis [107]. Finally, WTAs/LTAs have been shown to prevent autolysin binding on the bacterial surface, except to the cell septum, where these molecules are presumably absent [115].

In LAB, *L. lactis, L. plantarum*, and *L. rhamnosus dlt *mutants had faster autolysis rates than did wild-type strains, as a result of the activity of the major autolysins AcmA, Acm2, and Msp1, respectively [84,85,112]. In *L. lactis*, this phenotype was associated with a decreased degradation of AcmA by HtrA, cell wall-housekeeping protease [84].

#### Role of TAs in bacterial cell morphogenesis

WTAs were long considered to be essential molecules because deletions of genes related to the WTA biosynthesis pathway are lethal in *B. subtilis*. However, more recent reports have indicated that the lethal effects of the mutations were due either to the accumulation of toxic intermediates or to the sequestration of the undecaprenol phosphate carrier that is also required for PG synthesis [116] (Figure 2). This argument is supported by the fact that viable mutants lacking WTAs were obtained in *B. subtilis *and *S. aureus *by inactivating the *tagO *and *tarO *genes, respectively; they each encode the first enzyme of the biosynthesis pathway. As a result, WTAs are no longer considered as essential in *B. subtilis*, although their absence severely alters cell morphology and growth [117]. The role of teichoic acids in cell division and morphogenesis has been investigated in *B. subtilis*, and it appears that WTAs are involved in bacterial elongation, while LTAs participate in cellular division [118]. The concurrent absence of WTAs and LTAs is lethal for *B. subtilis*, which suggests that anionic polymers are a necessary component of Gram-positive cell walls. In *S. aureus*, WTAs have been described as acting as temporal and spatial regulators of PG cross-linking [119].

In *L. plantarum*, a *tagO *deletion mutant revealed that while WTAs are not essential for survival, they are required for proper cell elongation and cell division [120]. Atomic force microscopy (AFM) imaging of the bacterial cell surface combined with fluorescent labeling with lectin probes has revealed that WTAs exhibit a polarized distribution across the cell surface and that they are absent from the cell's poles. In addition, it appears that the polarized distribution of WTAs plays a key role in controlling cell morphogenesis (surface roughness, cell shape, and elongation and division) [120]. Furthermore, in *L. plantarum*, D-alanylation of LTAs plays an important role in cell morphology: the longer bacterial cells observed in the *dltD *mutant indicate that its elongation process is altered [85].

#### LTAs as bacteriophage receptors

LTAs have been shown to be receptor components for the bacteriophage LL-H that infects *Lactobacillus delbruekii *subsp. *lactis *ATCC15808 [121]. Moreover, D-Ala and α-Glc substituents of the poly(Gro-P) LTA backbone affect phage adsorption. A high degree of D-alanylation decreased adsorption, whereas Glc substituents were required for efficient binding, indicating that these LTA structural modifications affect how well the anti-receptor protein of the phage tail binds to LTAs [122].

#### Role of LTAs in bacteria-host cross-talk

LTAs appear to play a prominent role in host-lactobacilli interactions [101]. First, LTAs have been reported to be major players in *Lactobacillus johnsonii *La1 adhesion to human intestinal epithelial cells (Caco-2), possibly via hydrophobic interactions [123]. Also, TA D-Ala depletion can result in impaired colonization of the mouse gastrointestinal tract by *L. reuteri *[124].

Moreover, LTAs are MAMPs that bind to Toll-like receptor 2 (TLR2), a PRR that is present on the surface of epithelial and antigen-presenting cells and that, after being stimulated, can activate cytokine release [89]. It has been reported that LTAs purified from *L. casei *YIT 9029 and *L. fermentum *YIT 0159 significantly induce TNF-α secretion from murine macrophages, via a TLR2-mediated strain-dependent mechanism [125]. A *dlt *mutant of *L. plantarum *NCIMB8826--whose LTA D-alanylation was substantially reduced--exhibited anti-inflammatory properties, which contrasted with the properties demonstrated by the parental strain. In the mutant as compared to the wild type, the secretion of pro-inflammatory cytokines, such as TNFα and IL12, by peripheral blood monocyte-derived cells (PBMCs) was dramatically reduced and IL10 secretion was concurrently increased [100]. Moreover, the *dlt *mutant conferred protection against inflammation in a murine model of trinitrobenzene sulfonic acid (TNBS)-induced colitis. These results were confirmed using highly purified LTAs, which stimulated TLR2-dependent pro-inflammatory cytokine production [100]. In contrast, in *in vitro *studies, an *L. rhamnosus *GG *dltD *mutant, whose LTAs lacked D-alanyl esters, did not demonstrate significantly changed cytokine production [112]; however, it was associated with improvement in some colitic parameters in moderate to severe DSS-induced colitis in a murine model [126]. Furthermore, in an *Lactobacillus acidophilus ltaS *mutant deficient in LTAs, IL12 and TNF-α secretion in bone marrow-derived dendritic cells (DCs) was downregulated, while IL10 secretion and the expression of costimulatory molecules on the surfaces of the DCs were significantly enhanced. When mice with DSS-established colitis were treated with the *ltaS *mutant, their condition improved as a result of a mechanism involving IL10 and CD4+ FoxP3+ T-regulatory cells [127].

## Wall polysaccharides

### Structure and biosynthesis

Gram-positive cell walls frequently contain PSs in addition to PG and TAs. Bacterial PSs can be divided in three groups: exopolysaccharides (EPSs), which are loosely associated with the microbial cell surface and released into the surrounding environment; capsular polysaccharides (CPSs), which are permanently attached to the cell, forming a shield around the bacterium; and cell wall polysaccharides (WPSs), which may or may not be covalently attached to the cell wall but that do not form a capsule. It is worth noting that there is some controversy at the experimental level with regards to the definition of these three groups, and that the nomenclature is not strictly followed in the literature; for instance, EPS is also used to mean extracellular polysaccharide. Bacterial PSs exhibit great diversity, not only in sugar composition but also in linkage, branching, and substitution. We will not review here the *sensu stricto *EPSs that are released in the culture medium and that have been the subject of extensive research because of their role in adding texture to fermented milk products; they have been covered by other reviews [128,129]. We will only examine the PSs that have been found to be associated with bacterial cells.

The genes encoding molecules involved in PS biosynthesis are typically organized in clusters of 8 to 25 genes, which are located in the chromosome or plasmids. These clusters contain genes that encode glycosyltransferases and genes that are responsible for export, and regulation [130]. The PS synthesis pathway may overlap with those responsible for generating other cell wall polymers, such as PG or WTAs, since it may also involve undecaprenyl phosphate as a lipid carrier (Figure 2).

**Figure 4:**
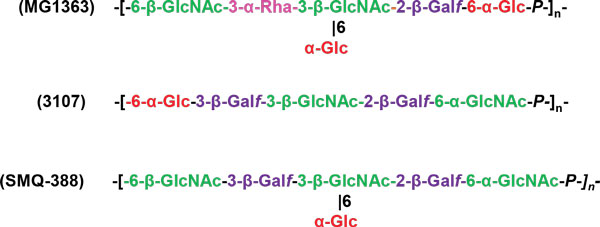
**Structure of the sugar-phosphate polysaccharide pellicle of three different *L. lactis *strains MG1363, 3107 and SMQ-388**.

In *L. lactis *MG1363, a WPS was discovered at the bacterial surface and subsequently characterized [131]. AFM as well as complementary transmission electron microscopy observations have shown that this WPS type forms a compact outer layer that surrounds the cell, named the pellicle. Its structure was established by NMR and is distinct from those of other bacterial PSs, including the previously characterized *L. lactis *EPS. The PS chains are composed of hexasaccharide-phosphate repeating units that contain rhamnose (Rha). They are likely covalently attached to the cell wall since they were only able to be extracted using a harsh acid treatment. In *L. lactis *MG1363, the molecules involved in PS synthesis are encoded by a single large cluster of genes. This gene cluster is found in many different *L. lactis *strains but exhibits a rather high level of genetic diversity, which suggests that there are structural variations in the WPSs synthesized by different *L. lactis *strains [132]. The structures of WPSs purified from two other *L. lactis *strains, 3107 and SMQ-388, were recently described and confirmed the PS structural diversity between *L. lactis *strains. Like the WPSs making up the MG1363 PS pellicle, these WPSs are acidic PSs made of oligosaccharide repeating units that are linked by phosphodiester bonds; however, the structure of the oligosaccharide repeating units differs among the three strains (Figure 4) [133,134].

HeteroPSs, which are composed of different sugar moieties (Glc, Gal, Rha, GlcNAc, and GalNAc), and other residues, such as glucuronic acid and Gro-3-P, have also been found to be associated with the cell surfaces of lactobacilli [135]. Notably, the *L. plantarum *WCFS1 genome contains four gene clusters associated with surface PS production [136]. The structure of these different PSs has not yet been determined. In *L. rhamnosus *GG, a long galactose-rich PS (named EPS--extracellular polysaccharide--by the authors) was detected at the bacterial surface using AFM [137,138]. It likely corresponds to a previously described PS structure [139]. *L. rhamnosus *strain GG and other strains show genetic differences in the gene cluster that encodes PS biosynthesis, differences that are linked to variation in PS composition [140]. Exploration of the *L. rhamnosus *GG cell surface using AFM revealed that its morphology is rough and characterized by wave-like structures [138]. In contrast, the cell surface of a PS-negative mutant was found to be much smoother, which suggests that the wave-like structures reflect PS production. Furthermore, single molecule force spectroscopy with lectin-modified tips revealed that cell surface PS chains had heterogeneous structures: there were PSs rich in mannose (or glucose) that had moderate extensions and PSs rich in galactose that had much longer extensions [138]. A lectin microarray developed to compare the surface glycomes of *L. casei *strains revealed that different strains had different profiles, which suggests that their WPSs are different [135]. In probiotic *L. casei *Shirota, two types of WPSs were found: longer, high molecular mass PS-1 and shorter low molecular mass PS-2. The structure of PS-1 has been described [141], and the gene cluster encoding proteins involved in PS-1 biosynthesis has been identified [142]. Furthermore, it was recently observed that *Lactobacillus helveticus *strains differ in WPS structure, and it has been hypothesized that these differences may partially explain variation in autolytic properties among the strains studied [143]. In conclusion, WPSs are omnipresent components of LAB cell surfaces, and it is likely that they differ structurally among strains of the same species.

### Functions of WPSs

A number of roles have already been assigned to WPSs in LAB in bacterial physiology as well as in interactions with bacteriophages or eukaryotic hosts (Table 2).

#### *WPSs as bacteriophage receptors in *L. lactis

In *L. lactis*, WPSs are now considered to be receptors for bacteriophages belonging to the 936 and P445 families, which means that they allow bacteriophage adsorption at the cell surface. An *L. lactis *MG1363 mutant that lacked a PS pellicle made of hexasaccharide subunits linked through phosphodiester bonds was shown to be resistant to the 936-bacteriophage sk1, which strongly suggests that this WPS could be the sk1 phage receptor [131]. Indeed, previous studies using transposon random mutagenesis mapped the genes required for the adsorption of two 936-type bacteriophages in their respective host strains; these genes were found inside gene clusters potentially implicated in WPS biosynthesis in *L. lactis *IL1403 and Wg2 [144] and that are homologous to the cluster that encodes the PS pellicle in MG1363. Recent research revealed a correlation between the pellicle genotype of a given *L. lactis *strain and the host range of these 936-type phages [132]. The findings support the PS pellicle's proposed role as a 936-phage receptor and suggest that variation in PS pellicle structure among strains could explain the narrow host range of this phage group. On the basis of bioinformatic analysis of the PS-encoding gene cluster, three major groups of *L. lactis *strains were distinguished (types A, B and C) [132]; more recently five subtypes (C1 to C5) could be identified in the C-group on the basis of differences in the variable region present in the C-type PS biosynthesis locus [133]. When genes from the variable region of the C2 subtype strain 3107 were expressed in a mutant of *L. lactis *NZ9000 of subtype C1 deficient in WPS synthesis, the resulting recombinant NZ9000 strain synthesizes WPS with the structure of subtype C2. In addition, by challenging the recombinant strain with bacteriophages infecting *L. lactis *3107, it was shown that WPS is the host cell surface receptor of the tested phages from 936 and P335 groups [133]. At the phage level, receptor-binding proteins (RBPs; also named anti-receptors) located at the tip of phage tail are involved in phage adsorption: they specifically recognize receptors on the bacterial surface. The 3D structures of RBPs in different lactococcal phages have been established, which means that the recognition mechanism that mediates interactions between RBPs and the PS pellicule can now be explored, with a view to understanding the molecular mechanisms underlying recognition specificity [145]. As a first step, surface-plasmon resonance experiments have demonstrated that bacteriophage p2 RBPs bind to PS pellicle purified from the phage's host strain--MG1363 [146].

#### Other functions of WPSs

An *L. lactis *MG1363 mutant that lacked surface WPSs produced long chains of unseparated cells that showed some morphological defects [131]. These observations suggest that WPSs are required for normal cell morphology and that they play a role in cell division.

Additionally, surface-exposed PSs are involved in a wide range of bacterial properties and functions, including adhesion to abiotic surfaces and biofilm formation; they also participate in interactions with other microorganisms and host cells. Inactivation of the glycosyltransferase *welE *gene in *L. rhamnosus *GG greatly reduced levels of high molecular mass, galactose-rich WPSs [137]. The *welE *mutant exhibited increased adherence and a greater capacity to form biofilms, possibly because surface adhesins, such as pili structures, were more exposed.

Bacterial CPSs have been shown to be potent immunomodulating molecules; they have largely been characterized in pathogenic species [130] and are considered to be virulence factors that act by preventing phagocytosis. The *L. lactis *PS pellicle has also been shown to protect bacteria against phagocytosis by murine macrophages *in vitro *[131], which suggests that WPSs may shield other cell surface components and prevent them from being recognized by macrophage receptors. An *L. casei *Shirota mutant that produced lower levels of high molecular mass WPSs generated higher levels of IL6, IL10, and IL12 cytokines after being co-incubated with murine macrophages *in vitro*. These results highlight the immunosuppressive function of WPSs [142]. Similar results were obtained with *L. plantarum *mutant produced by deleting the four gene clusters that encode the proteins that make up the PS biosynthesis pathways. The mutant as compared to the wild type, elicited a dramatic increase of TLR2-mediated NF-κB activation which suggests that the presence of surface PSs reduces the exposure of TLR2-activating molecules [136]. Finally, the galactose-rich PSs of *L. rhamnosus *GG confer protection against host innate defense molecules, such as the LL-37 antimicrobial peptide [147].

## Cell wall proteins

### Different modes of attachment to the cell wall

After being synthesized in the cytoplasm, 5-10% of bacterial proteins are released outside the cytoplasmic membrane [148]. In Gram-positive bacteria, most of these proteins are secreted by the universally conserved and essential Sec pathway. This pathway has been extensively studied in *E. coli*, and genome analyses have revealed that homologs exist in other bacteria, including LAB [149]. Almost all proteins that are targeted by this secretory pathway have an N-terminal signal peptide composed of approximately 30 amino acids. Once the proteins have been translocated across the cytoplasmic membrane, this signal peptide is cleaved off by the appropriate signal peptidase. Then, the protein is either released into the extracellular medium or, alternatively, it is retained in the cell envelope, if it contains a specific sequence ensuring its attachment to the cytoplasmic membrane or the components of the cell wall in addition to the signal peptide. In LAB, surface-associated proteins make up around 80% of predicted secreted proteins [148]. Secreted proteins can be covalently attached to the cell surface by sortase-mediated reactions or non-covalently attached via i) transmembrane anchors; ii) lipid anchors; or iii) different cell wall binding domains (CWBD) [150,151]. We will review here LAB proteins which are linked to cell wall components through covalent or non covalent binding.

#### PG-anchored proteins

A portion of a given cell wall protein is covalently bound to PG by a transpeptidation mechanism that is catalyzed by sortase A (SrtA, also called housekeeping sortase). In addition to an N-terminal signal peptide, they also contain, at their C-terminal, a conserved LPXTG motif that is followed by a stretch of hydrophobic residues and a positively charged tail [149,152,153]. Transpeptidase SrtA, which is located in the membrane, cleaves the Thr-Gly bond of the LPXTG motif and links the Thr carboxyl group to the free amino group of the side chain of the lipid II PG precursor. The presence of SrtA and LPXTG-containing proteins is well documented in pathogens such as *S. aureus *[154], *E. faecalis, E. faecium *[155], and *L. monocytogenes *[156]. This SrtA-specific mode of protein attachment to PG is characteristic of all Gram-positive bacteria, including LAB [157]. Inactivation of *srtA *in *L. lactis *IL1403 has demonstrated that this gene is responsible for the cell wall anchoring of at least five LPXTG-containing proteins [158].

One remarkable family of LPXTG proteins found in LAB is the one of mucus-binding proteins. These proteins contain mucus-binding domains (MUB or MucBP) that are thought to play an important role in the adhesion of LAB to the mucus layer that covers intestinal epithelial cells [159]. Other functionally important LPXTG proteins are the pilins, which are the structural components of pili. Pili (or fimbriae) are long filamentous structures that extend from the surfaces of various Gram-negative and Gram-positive bacteria. Most studies on pili in Gram-positive bacteria have been conducted on pathogenic species, including streptococci, enterococci, corynebacteria, and bacilli [160-162]. Pili have been shown to be involved in adhesion to host cells and tissues and are thus considered to promote host colonization and invasion [162]. In Gram-positive bacteria, the sortase-dependent pili (Spa-type for sortase-mediated pilus assembly) are composed of a major backbone pilin, whose subunits are covalently assembled by sortase C, and of one or two accessory pilins. The minor pilins are located at the base and the tip of the pilus and are possibly also dispersed along the shaft. The pili structures are anchored to PG by housekeeping sortase A [161]. The presence of pili in LAB and in bifidobacteria has also been described and has been linked to the ability of these bacteria to colonize the guts of their hosts and persist in their gastrointestinal tracts [163,164]. *L. rhamnosus *GG cells have been found to contain multiple pili (an average of 10-50 per cell) with lengths of up to 1 μm that are predominantly located near the cell poles [165]. More recently, *L. lactis *surface pili were visualized using electron microscopy and AFM [166,167]. In a natural *L. lactis *isolate, a plasmid-encoded pilin gene cluster that encodes sortase-dependent pili was shown to be responsible for the assembly of surface pili [167]. This strain produces thin pili that are rather short (averaging 350 nm length).

#### Proteins that are noncovalently bound to the cell wall

These proteins contain specific CWBDs that have been described in several reviews [151,168]. Here we focus on CWBDs that are found in LAB and their bacteriophages.

#### LysM domain

The LysM sequence **(**Lys motif, PF01476**) **is about 40 amino acid residues long and is present in more than 2,000 eukaryotic and prokaryotic proteins. Several LysM sequences linked by intervening sequences constitute a LysM domain [70,168,169]. Studies examining the binding patterns of different PG chemotypes have found that LysM non-covalently binds to the GlcNAc moiety of glycan chains [70]. However, binding is not disrupted by *N*-deacetylation of GlcNAc [54] or by *O*-acetylation of MurNAc [170].

LysM domains are most commonly found in the N-terminal or C-terminal regions of PGHs [168,171] and contain one to six LysM sequences. The presence of an optimal number of LysM sequences is crucial for the enzymatic activity of PGHs and, as a consequence, for the different functions of these bacterial enzymes: they are involved in cell growth, cell separation, and autolysis. The main lactococcal autolysin AcmA, which is one of the best studied PGHs, has a modular structure and a C-terminal LysM domain that contains three LysM sequences and an N-terminal N-acetyl-glucosaminidase catalytic domain [68]. All three LysM sequences are required for AcmA to function optimally [69], but a single LysM suffices for PG binding [172]. AcmA also binds to PG in other bacteria, and even to the cells of different Gram-positive species in mixed communities [173].

It has been shown that, in *L. lactis*, the AcmA LysM domain binds near the cell poles and the cell septum [70]. At the surface of Gram-positive bacteria, the binding of LysM-containing proteins may be hindered by CW polymers, such as WPSs or WTAs, which results in localization of PGHs in the septal region of the cell [169]. In the case of the lactococcal autolysin AcmA, it has been proposed that attachment to the cell wall can be hindered by CW constituents; LTAs are suggested candidates [70,174]. Another possible candidate is the surface PS pellicle: using AFM and employing tips coated with the AcmA-derived LysM domain, *L. lactis *PS pellicle was found to be capable of blocking the binding of LysM to PG [39,175].

#### *Bacterial SH3b domain *(including the subfamilies SH3_3, SH3_4, and SH3_5)

This domain is the bacterial equivalent of the well-characterized SH3 domain that is found in eukaryotes and viruses. Conflicting results have been obtained when it comes to the PG motif recognized by this domain. In staphylococci with a five-Gly PG crossbridge, the length and amino acid composition of the cross-bridge have been found to have a significant impact on the binding of the SH3-containing homolog of lysostaphin ALE-1 [176]. Also, it has been proposed that the C-terminal domain of lysostaphin, which contains the SH3_5 domain, directs the enzyme to the cross-linked PG [177]. However, more recently, single-molecule AFM experiments using tips functionalized with the *L. plantarum *Acm2 (which contains five SH3_5 domains) have found that SH3b domains bind to PG glycan chains and that the binding site contains GlcNAc [178].

#### WxL domain

This domain was initially identified based on *in silico *analysis of gene clusters that encode the cell surface proteins of lactobacilli, enterococcoci, and listeria species [179]. Proteins containing the WxL domain have been experimentally shown to non-covalently bind to PG in *E. faecalis *[180]. Proteins containing the WxL domain are present in *L. plantarum *(19), *L. sakei *(15), *L. lactis *(7), *L. casei*, and *Lactobacillus coryniformis *(1) [180]; however, their functions have not yet been identified.

#### Lc-LysBD domain

This domain was recently discovered in the C-terminal of the endolysins (Lc-Lys and Lc-Lys2) of prophages found in the complete genome sequence of *L. casei *BL23 [181]. It does not share amino acid sequence similarity with any known CWBDs. The domain can bind to PG and can specifically recognize the amidated D-Asp cross-bridge that occurs in *L. casei *PG (Figure 1). Remarkably, it does not bind to PG molecules with non-amidated D-Asp cross-bridges or PG molecules with different types of cross-bridges, such as the L-Ala-L-Ala/L-Ser bridge. This domain is also present in the endolysins of other *L. casei *phages--A2 and PL-1--and in the endolysin of *L. lactis *phage 949 [182].

#### SLH-domains

The surface (S) layer entirely coats the bacterial surface and is composed of (glyco)proteins that intrinsically form a two-dimensional paracrystalline structure. Most prokaryotic S-layer proteins possess a signal peptide. These proteins bind non-covalently via their N- or C-terminus to PG or secondary cell wall polymers. The attachment is mediated by S-layer homologous domains (SLHDs), which can also be found in other enzymes of Gram-positive bacteria [149,183]. Most often, S-layer proteins possess three SLHDs, each consisting of 50-70 amino acids. S-layer proteins are present in lactobacilli, and their structure and functions have already been extensively reviewed [184]. The cell wall ligands of the S-layer proteins isolated from different *Lactobacillus *species have been proposed to be carbohydrates either teichoic acids or neutral polysaccharides [184].

### Role and applications of cell wall proteins

#### Role of cell wall proteins in bacteria-host interactions

The surface proteins of probiotic or commensal bacteria are thought to facilitate mucosal colonization and persistence in the gastrointestinal tract; they may also favor cross-talk with immune cells by mediating direct contact with the intestinal mucosa. The role of pili appendages and mucus-binding proteins as surface determinants in certain LAB strains has been underscored: they allow bacteria to adhere to intestinal epithelial cells or mucus. Notably, the pili identified in *L. rhamnosus *GG confer the ability to adhere to the intestinal epithelial cells (Caco-2) and human intestinal derived mucus; they also promote biofilm formation [185,186]. Furthermore, pili synthetized by a natural isolate of *L. lactis *allow the strain to adhere to intestinal epithelial cells (Caco-2) [167]. Two types of surface determinants--pili and mucus-binding proteins--have also been shown to play a role in bacterial adhesion to model mucins, and mucus-binding proteins make a greater contribution under shear flow conditions [187]. Moreover, *L. rhamnosus *GG pili are involved in reducing IL8 mRNA expression provoked by other cell surface components, such as LTAs in intestinal epithelial cells [185]. Other cell wall-associated or secreted proteins of probiotic strains have also been shown to be involved in modulating the response of the host immune system. In *L. acidophilus *NCFM, the S-layer protein A (SlpA) has been found to be a ligand that is recognized by the surface lectin receptor DC-SIGN, which is functionally involved in the modulation of DCs [188]. The attachment of bacteria to DCs has been shown to stimulate immature DCs and regulate T-cell function. Also, p40 and p75, two proteins described above (section 1.6) that demonstrate PGH activity and that are secreted by *L. rhamnosus*, prevent cytokine-induced apoptosis in IECs, and anti-inflammatory properties have been attributed to the action of p40's N-terminal [92-94].

#### Applications for vaccine development

From an applied perspective, LAB, because of their GRAS status, are considered to be convenient vectors for delivering therapeutic proteins or antigens to gastrointestinal tract mucosa. In an alternative approach to vector creation that avoids the use of genetically modified bacteria, proteins of interest can be fused with CWBDs found in cell wall proteins and then anchored on the surfaces of LAB. The ability of LysM and SLH domains to bind to bacterial cell walls has been exploited to display protein antigens on LAB surfaces when developing oral vaccines [184,189].

## Concluding remarks and perspectives

Remarkable advances have been made in the last two decades in terms of understanding the structure and function of LAB cell walls. Cell wall components have been purified from several LAB species, which has allowed the elucidation of fine-scale cell wall structure as well as interspecific and intraspecific variation. In tandem, genes involved in cell wall biosynthesis, modification, and degradation pathways have been identified, which has allowed for the construction of mutants that can be used to investigate the role of such genes in bacterial physiology; the results obtained can also inform technological and health applications of LAB. Specific progress has been made with regards to deciphering the molecular mechanisms that control PGH activity and bacterial autolysis, the anchoring of cell wall proteins on the bacterial surface, the adsorption of bacteriophages to the target bacterial surface, and the cross-talk between probiotic bacteria and host cells. The results obtained have underscored the importance of further investigating LAB cell wall structure and function and thus expanding into new directions of research.

Novel structural modifications of PG have been identified, along with the genes that are involved. However, the role of these modifications in bacterial physiology, their distribution along the inner cell wall, and their influence on bacteria-host interactions remain to be investigated in detail. Furthermore, the enzymes responsible for PG modifications, such as *O*-acetyltransferase OatA [44], may be working in concert with other proteins that are involved in cell division, proteins that remain to be identified.

*O*-glycosylation of PGHs has been reported in LAB and has been found to modulate the PG-hydrolyzing activity of *L. plantarum*'s major autolysin Acm2. The function of such modifications needs to be investigated further in other LAB species and its role in bacteria-host interactions should be characterized.

Among the secondary cell wall polymers that decorate the PG sacculus are WTAs. However, WPSs are also present, and they are essential for the proper septation and division of bacterial cells, which indicates that they probably play a crucial role in maintaining cell wall architecture and integrity. Nonetheless, their exact function has not yet been deciphered. Further work should aim to identify both WPS binding sites on PG as well as the enzymes involved in creating the covalent bonds. The full range of WPS activity and the control that these molecules exert over cell wall protein localization also require further investigation.

The arrangement of the different polymers inside the cell wall remains largely unknown. AFM has already proven to be a powerful technique with which to explore bacterial surface architecture at the nanoscale. Topographic imaging of the surface of several LAB, including *L. lactis, L. plantarum*, and *L. rhamnosus *[175], has provided very high resolution images of the bacterial cell surface structures (e.g., PG, TAs, PS, and pili) present on living cells, all without provoking denaturation. In addition, single-molecule force spectroscopy may be used to explore the spatial distribution and molecular elasticity of such structures.

The structural diversity that exists in cell wall components among bacterial species and strains may underlie strain-dependent differences in processes such as autolysis and characteristics such as stress resistance, probiotic properties, or phage sensitivity; consequently, this diversity merits further study. For instance, a better understanding of interstrain structural variation in *L. lactis *WPSs combined with the characterization of the 3D structure of phage RBPs should allow researchers to unravel the molecular interactions that take place between RBPs and WPS receptors. From an applied perspective, a more thorough comprehension of the molecular mechanisms behind phage adsorption on host bacteria should allow us to design practical strategies to fight phage infections.

A first series of successes has stemmed from the characterization of the cell wall determinants involved in interactions between probiotic bacteria and host cells. The next step is to identify the host cell receptors that are involved in the recognition of cell wall components and the signal transduction pathways that lead to cell response. The identification of the active compounds found in probiotic bacteria could lead to the development of disease treatment strategies, as in the case of inflammatory bowel diseases, for instance.

## List of abbreviations used

LAB, lactic acid bacteria; GRAS, generally recognized as safe; PG, peptidoglycan; TA, teichoic acid; PS, polysaccharide; MurNAc, N-acetyl-muramic acid; GlcNAc, N-acetyl-glucosamine; WTA, wall teichoic acid; LTA, lipoteichoic acid; UDP, uridine diphosphate; PBP, penicillin-binding protein; PGH, peptidoglycan hydrolase; TCS, two-component system; Gro-P, glycerol-phosphate; Rbo-P, ribitol phosphate; CDP, cytidine diphosphate; LCP family, LytR-CpsA-Psr family; DC, dendritic cell; EPS, exopolysaccharide; CPS, capsular polysaccharide; WPS, wall polysaccharide; RBP, receptor binding protein.

## Competing interests

The authors declare that they have no competing interests.
